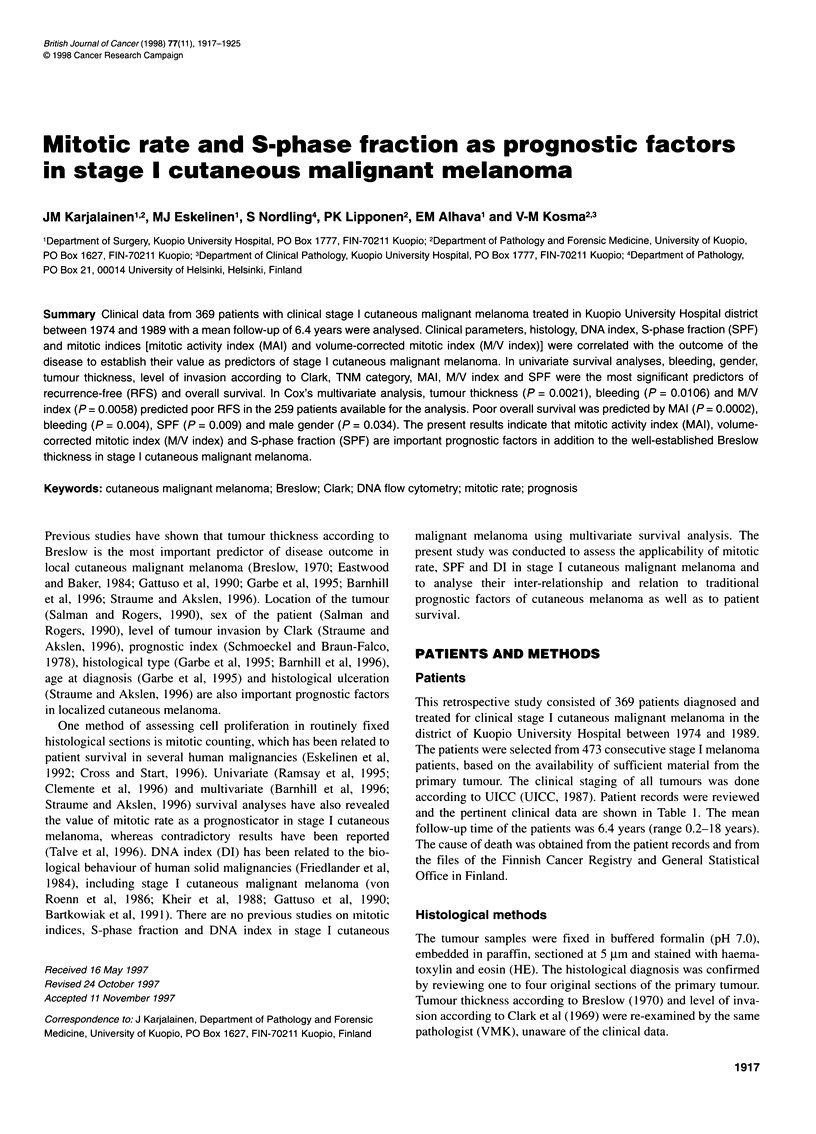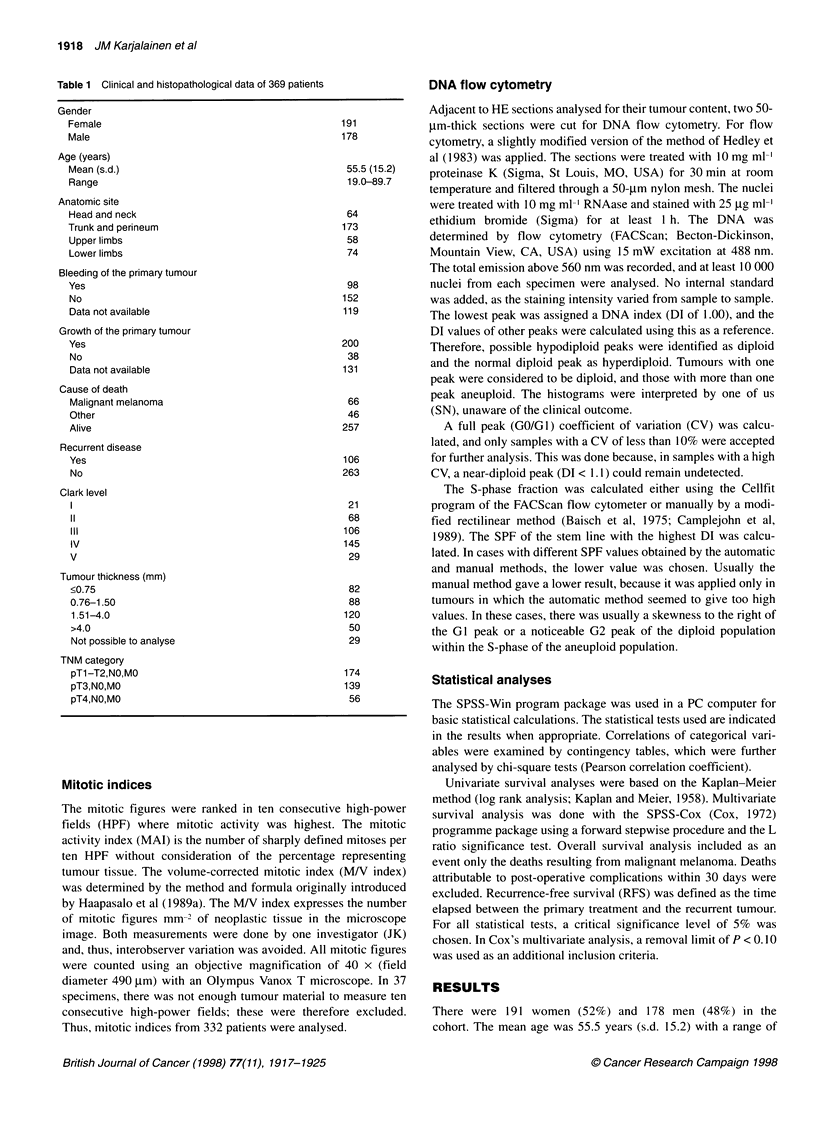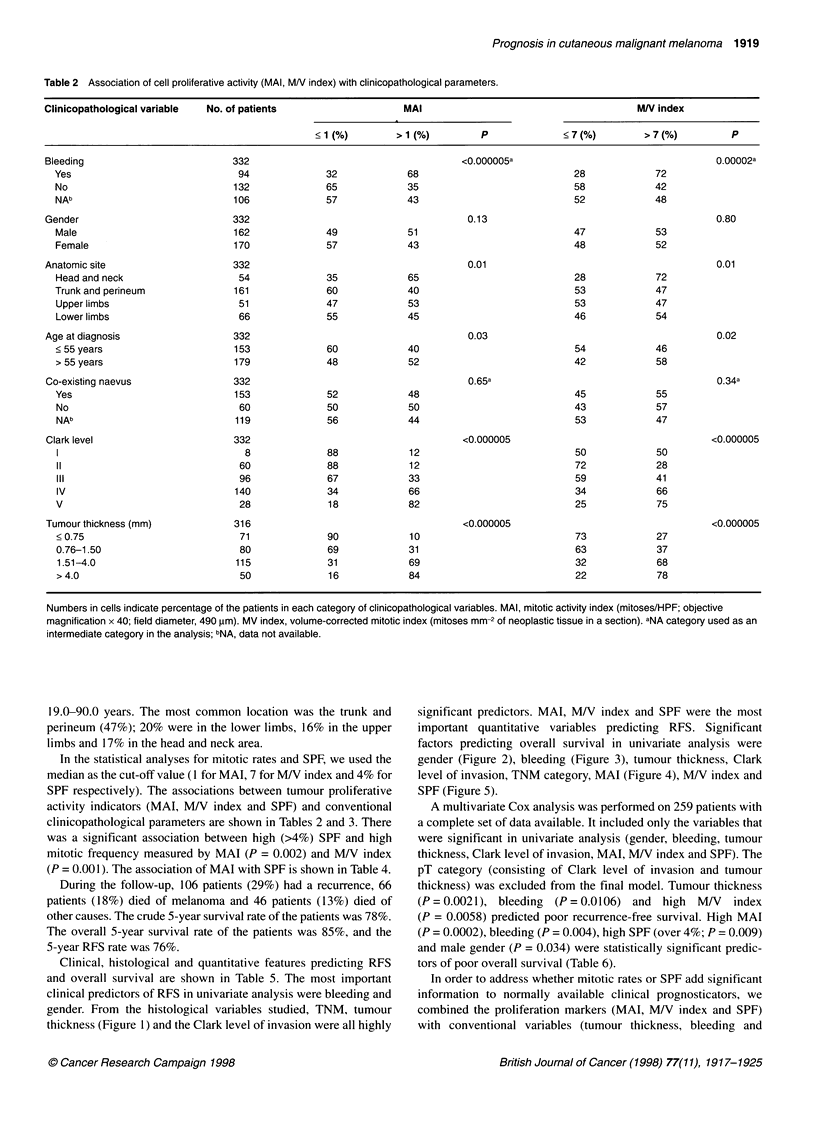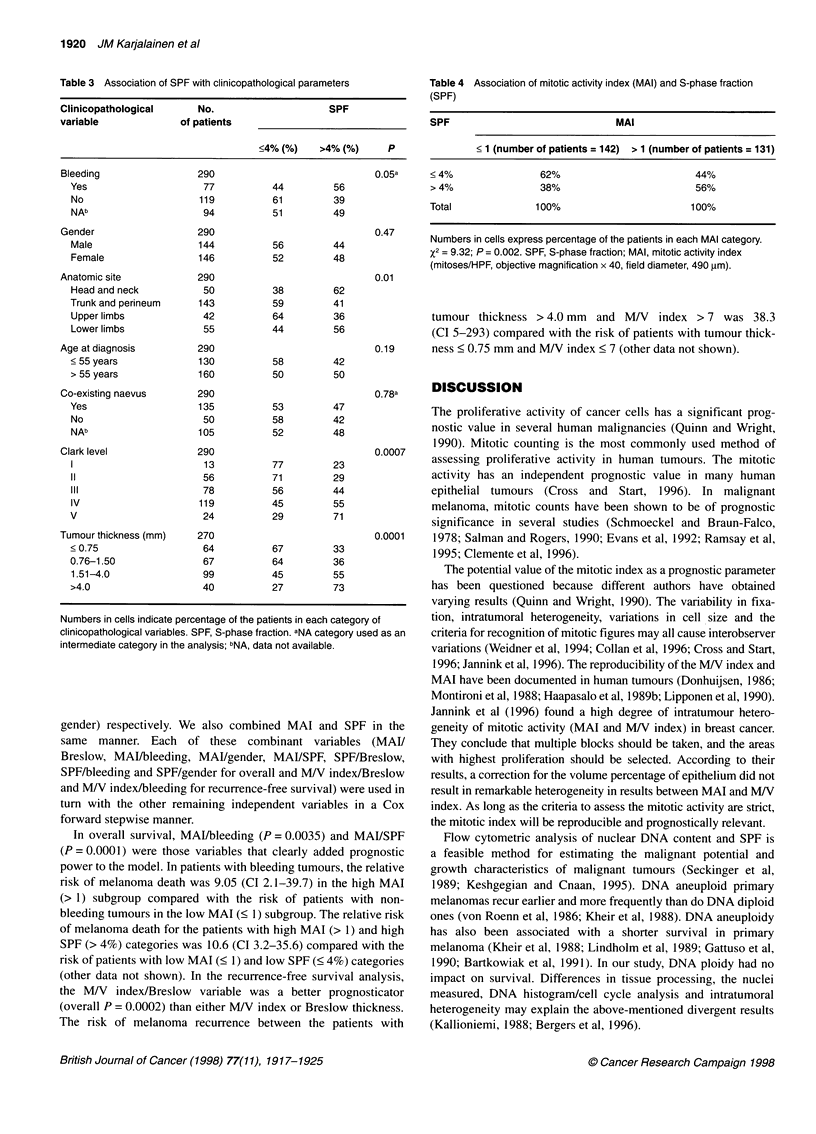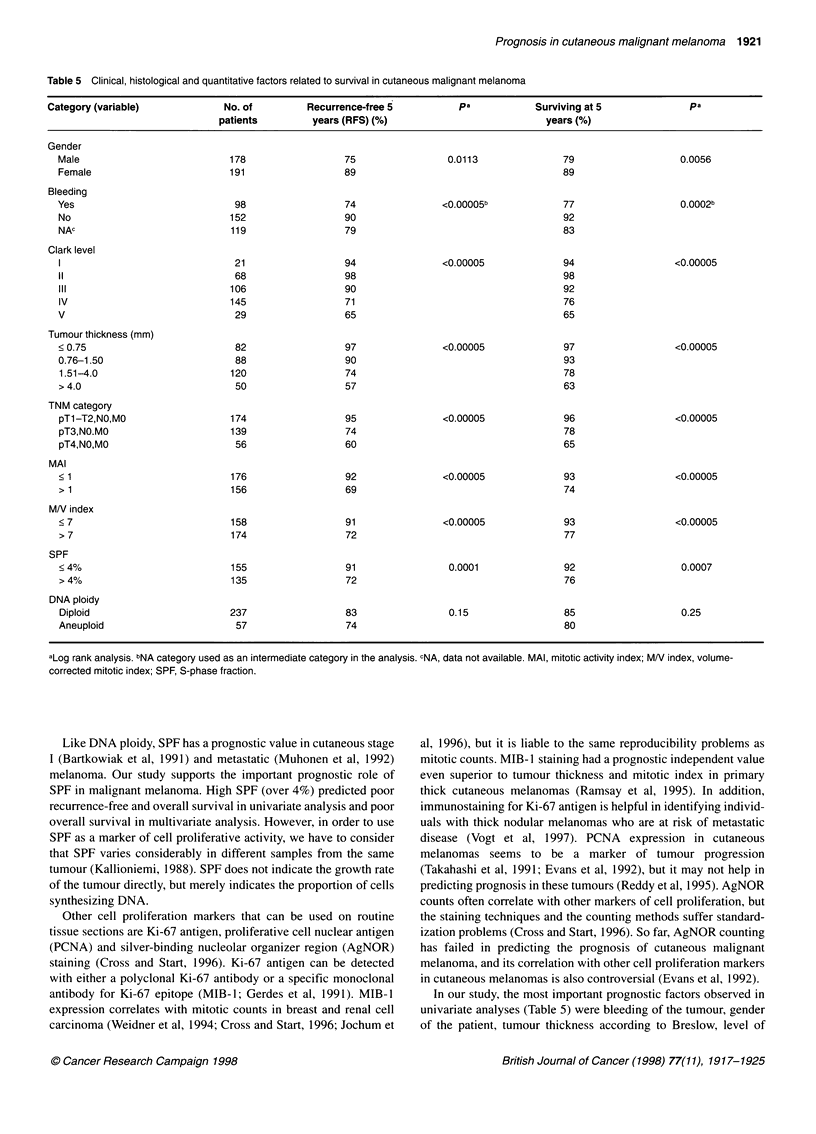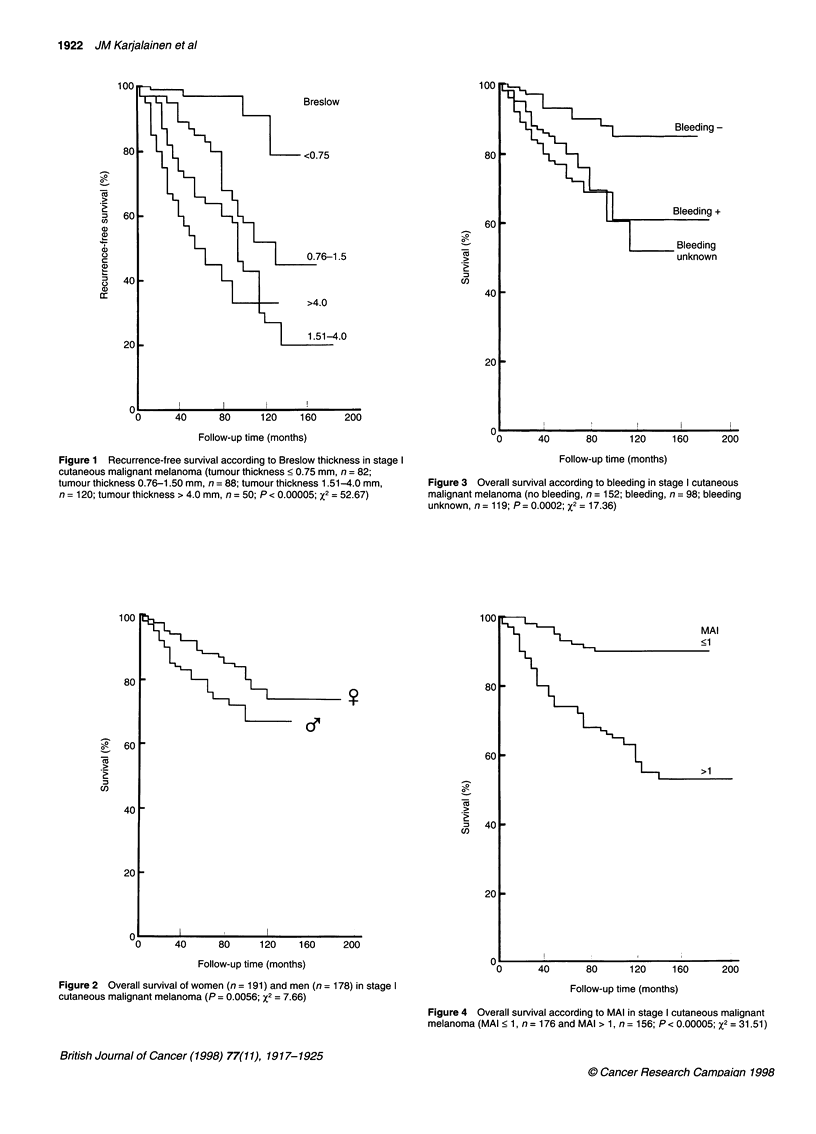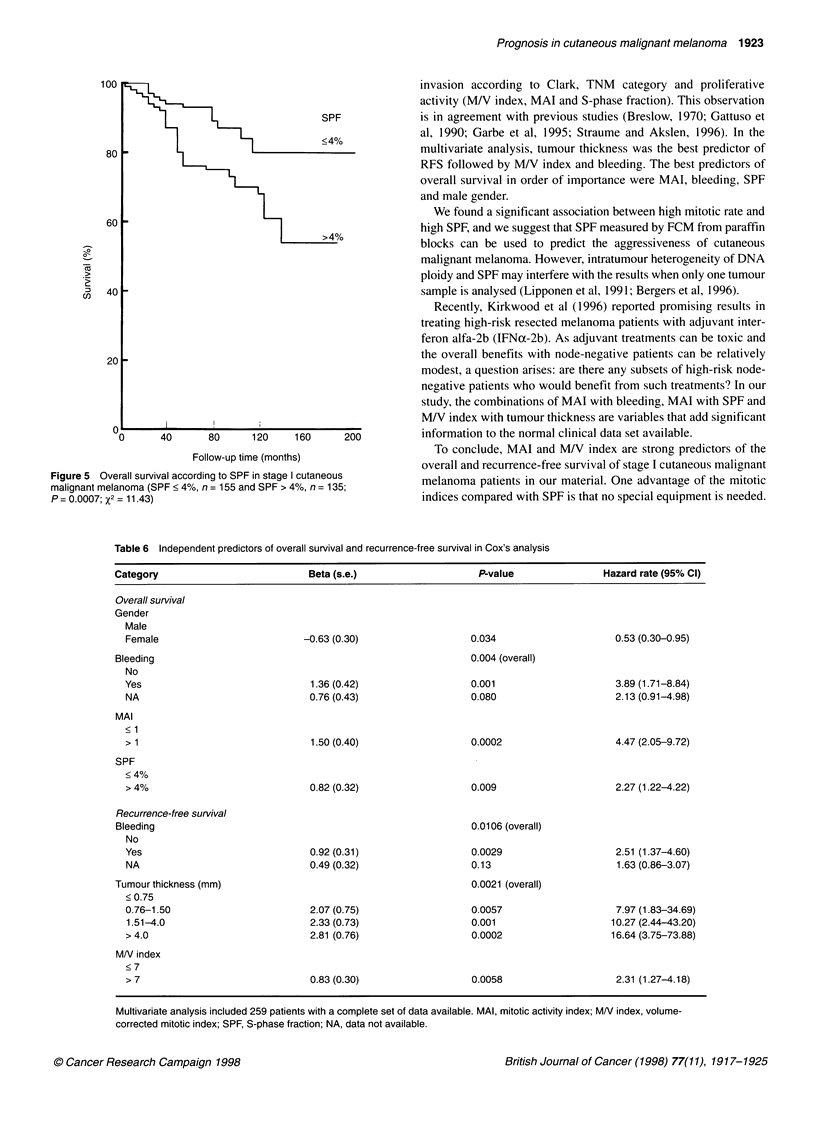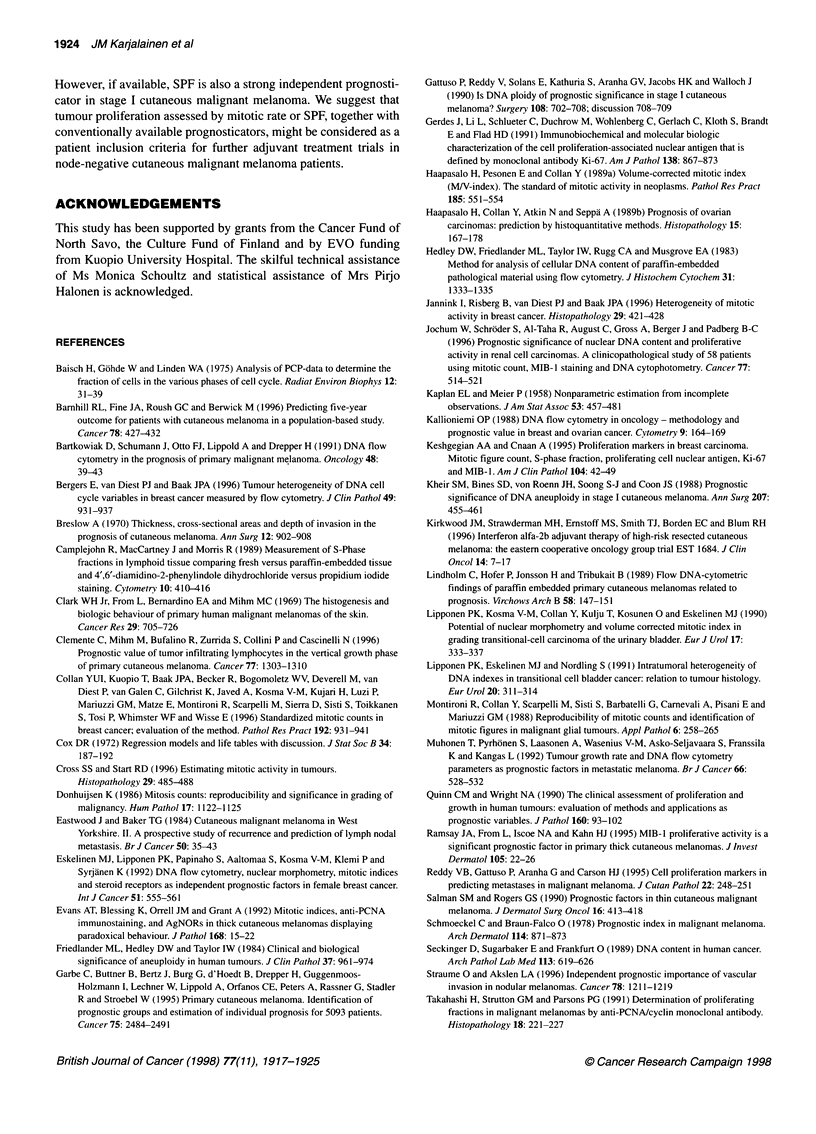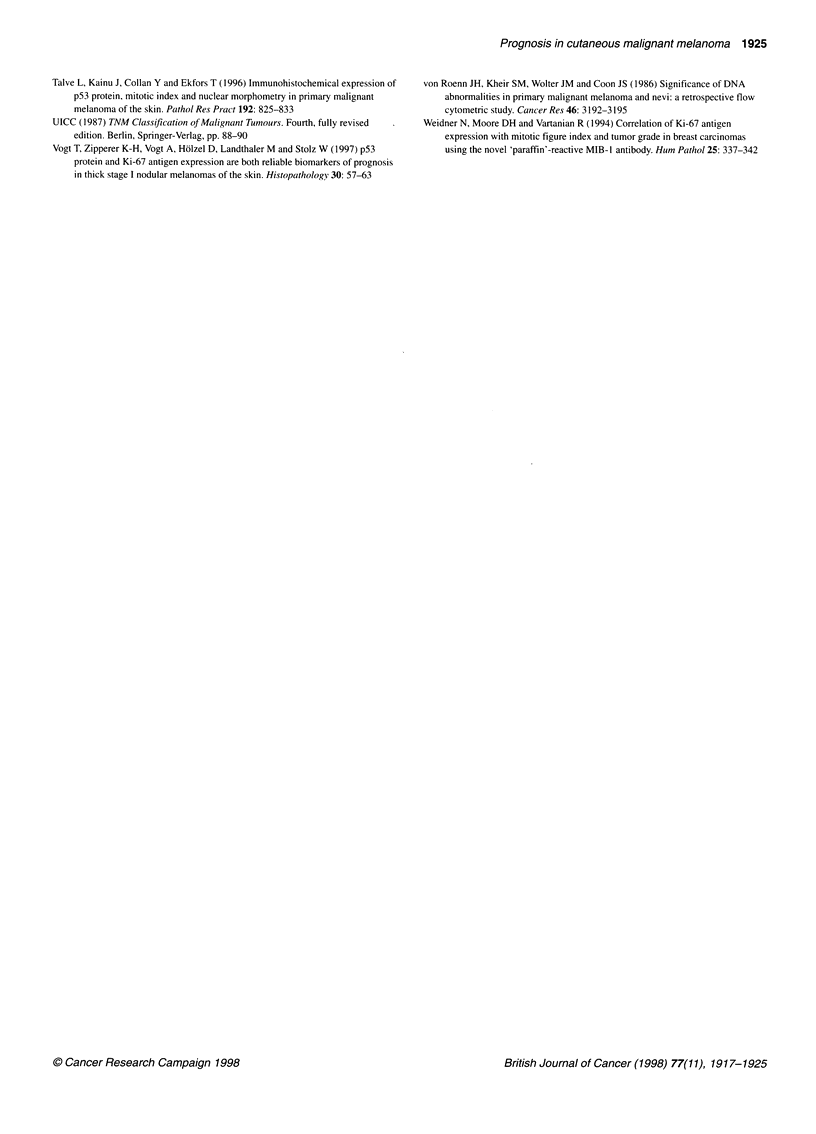# Mitotic rate and S-phase fraction as prognostic factors in stage I cutaneous malignant melanoma.

**DOI:** 10.1038/bjc.1998.318

**Published:** 1998-06

**Authors:** J. M. Karjalainen, M. J. Eskelinen, S. Nordling, P. K. Lipponen, E. M. Alhava, V. M. Kosma

**Affiliations:** Department of Surgery, Kuopio University Hospital, Finland.

## Abstract

Clinical data from 369 patients with clinical stage I cutaneous malignant melanoma treated in Kuopio University Hospital district between 1974 and 1989 with a mean follow-up of 6.4 years were analysed. Clinical parameters, histology, DNA index, S-phase fraction (SPF) and mitotic indices [mitotic activity index (MAI) and volume-corrected mitotic index (M/V index)] were correlated with the outcome of the disease to establish their value as predictors of stage I cutaneous malignant melanoma. In univariate survival analyses, bleeding, gender, tumour thickness, level of invasion according to Clark, TNM category, MAI, M/V index and SPF were the most significant predictors of recurrence-free (RFS) and overall survival. In Cox's multivariate analysis, tumour thickness (P = 0.0021), bleeding (P = 0.0106) and M/V index (P = 0.0058) predicted poor RFS in the 259 patients available for the analysis. Poor overall survival was predicted by MAI (P = 0.0002), bleeding (P = 0.004), SPF (P = 0.009) and male gender (P = 0.034). The present results indicate that mitotic activity index (MAI), volume-corrected mitotic index (M/V index) and S-phase fraction (SPF) are important prognostic factors in addition to the well-established Breslow thickness in stage I cutaneous malignant melanoma.


*British Journal of Cancer* (1998) **77**(11), 1917–1925
© 1998 Cancer Research Campaign

### Mitotic rate and S-phase fraction as prognostic factors in stage I cutaneous malignant melanoma

**JM Karjalainen[1,2], MJ Eskelinen[1], S Nordling[4], PK Lipponen[2], EM Alhava[1] and V-M Kosma[2,3]**

[1]Department of Surgery, Kuopio University Hospital, PO Box 1777, FIN-70211 Kuopio; [2]Department of Pathology and Forensic Medicine, University of Kuopio, PO Box 1627, FIN-70211 Kuopio; [3]Department of Clinical Pathology, Kuopio University Hospital, PO Box 1777, FIN-70211 Kuopio; [4]Department of Pathology, PO Box 21, 00014 University of Helsinki, Helsinki, Finland

**Summary** Clinical data from 369 patients with clinical stage I cutaneous malignant melanoma treated in Kuopio University Hospital district between 1974 and 1989 with a mean follow-up of 6.4 years were analysed. Clinical parameters, histology, DNA index, S-phase fraction (SPF) and mitotic indices [mitotic activity index (MAI) and volume-corrected mitotic index (M/V index)] were correlated with the outcome of the disease to establish their value as predictors of stage I cutaneous malignant melanoma. In univariate survival analyses, bleeding, gender, tumour thickness, level of invasion according to Clark, TNM category, MAI, M/V index and SPF were the most significant predictors of recurrence-free (RFS) and overall survival. In Cox's multivariate analysis, tumour thickness ($P$ = 0.0021), bleeding ($P$ = 0.0106) and M/V index ($P$ = 0.0058) predicted poor RFS in the 259 patients available for the analysis. Poor overall survival was predicted by MAI ($P$ = 0.0002), bleeding ($P$ = 0.004), SPF ($P$ = 0.009) and male gender ($P$ = 0.034). The present results indicate that mitotic activity index (MAI), volume-corrected mitotic index (M/V index) and S-phase fraction (SPF) are important prognostic factors in addition to the well-established Breslow thickness in stage I cutaneous malignant melanoma.

**Keywords:** cutaneous malignant melanoma; Breslow; Clark; DNA flow cytometry; mitotic rate; prognosis

Previous studies have shown that tumour thickness according to Breslow is the most important predictor of disease outcome in local cutaneous malignant melanoma (Breslow, 1970; Eastwood and Baker, 1984; Gattuso et al, 1990; Garbe et al, 1995; Barnhill et al, 1996; Straume and Akslen, 1996). Location of the tumour (Salman and Rogers, 1990), sex of the patient (Salman and Rogers, 1990), level of tumour invasion by Clark (Straume and Akslen, 1996), prognostic index (Schmoeckel and Braun-Falco, 1978), histological type (Garbe et al, 1995; Barnhill et al, 1996), age at diagnosis (Garbe et al, 1995) and histological ulceration (Straume and Akslen, 1996) are also important prognostic factors in localized cutaneous melanoma.

One method of assessing cell proliferation in routinely fixed histological sections is mitotic counting, which has been related to patient survival in several human malignancies (Eskelinen et al, 1992; Cross and Start, 1996). Univariate (Ramsay et al, 1995; Clemente et al, 1996) and multivariate (Barnhill et al, 1996; Straume and Akslen, 1996) survival analyses have also revealed the value of mitotic rate as a prognosticator in stage I cutaneous melanoma, whereas contradictory results have been reported (Talve et al, 1996). DNA index (DI) has been related to the biological behaviour of human solid malignancies (Friedlander et al, 1984), including stage I cutaneous malignant melanoma (von Roenn et al, 1986; Kheir et al, 1988; Gattuso et al, 1990; Bartkowiak et al, 1991). There are no previous studies on mitotic indices, S-phase fraction and DNA index in stage I cutaneous malignant melanoma using multivariate survival analysis. The present study was conducted to assess the applicability of mitotic rate, SPF and DI in stage I cutaneous malignant melanoma and to analyse their inter-relationship and relation to traditional prognostic factors of cutaneous melanoma as well as to patient survival.

*Received 16 May 1997*
*Revised 24 October 1997*
*Accepted 11 November 1997*

*Correspondence to:* J Karjalainen, Department of Pathology and Forensic Medicine, University of Kuopio, PO Box 1627, FIN-70211 Kuopio, Finland

### PATIENTS AND METHODS

### Patients

This retrospective study consisted of 369 patients diagnosed and treated for clinical stage I cutaneous malignant melanoma in the district of Kuopio University Hospital between 1974 and 1989. The patients were selected from 473 consecutive stage I melanoma patients, based on the availability of sufficient material from the primary tumour. The clinical staging of all tumours was done according to UICC (UICC, 1987). Patient records were reviewed and the pertinent clinical data are shown in Table 1. The mean follow-up time of the patients was 6.4 years (range 0.2–18 years). The cause of death was obtained from the patient records and from the files of the Finnish Cancer Registry and General Statistical Office in Finland.

### Histological methods

The tumour samples were fixed in buffered formalin (pH 7.0), embedded in paraffin, sectioned at 5 μm and stained with haematoxylin and eosin (HE). The histological diagnosis was confirmed by reviewing one to four original sections of the primary tumour. Tumour thickness according to Breslow (1970) and level of invasion according to Clark et al (1969) were re-examined by the same pathologist (VMK), unaware of the clinical data.

**Table 1** Clinical and histopathological data of 369 patients

| | |
|---|---|
| Gender | |
| Female | 191 |
| Male | 178 |
| Age (years) | |
| Mean (s.d.) | 55.5 (15.2) |
| Range | 19.0–89.7 |
| Anatomic site | |
| Head and neck | 64 |
| Trunk and perineum | 173 |
| Upper limbs | 58 |
| Lower limbs | 74 |
| Bleeding of the primary tumour | |
| Yes | 98 |
| No | 152 |
| Data not available | 119 |
| Growth of the primary tumour | |
| Yes | 200 |
| No | 38 |
| Data not available | 131 |
| Cause of death | |
| Malignant melanoma | 66 |
| Other | 46 |
| Alive | 257 |
| Recurrent disease | |
| Yes | 106 |
| No | 263 |
| Clark level | |
| I | 21 |
| II | 68 |
| III | 106 |
| IV | 145 |
| V | 29 |
| Tumour thickness (mm) | |
| ≤0.75 | 82 |
| 0.76–1.50 | 88 |
| 1.51–4.0 | 120 |
| >4.0 | 50 |
| Not possible to analyse | 29 |
| TNM category | |
| pT1–T2,N0,M0 | 174 |
| pT3,N0,M0 | 139 |
| pT4,N0,M0 | 56 |

### Mitotic indices

The mitotic figures were ranked in ten consecutive high-power fields (HPF) where mitotic activity was highest. The mitotic activity index (MAI) is the number of sharply defined mitoses per ten HPF without consideration of the percentage representing tumour tissue. The volume-corrected mitotic index (M/V index) was determined by the method and formula originally introduced by Haapasalo et al (1989a). The M/V index expresses the number of mitotic figures $mm^{-2}$ of neoplastic tissue in the microscope image. Both measurements were done by one investigator (JK) and, thus, interobserver variation was avoided. All mitotic figures were counted using an objective magnification of 40 × (field diameter 490 μm) with an Olympus Vanox T microscope. In 37 specimens, there was not enough tumour material to measure ten consecutive high-power fields; these were therefore excluded. Thus, mitotic indices from 332 patients were analysed.

### DNA flow cytometry

Adjacent to HE sections analysed for their tumour content, two 50-μm-thick sections were cut for DNA flow cytometry. For flow cytometry, a slightly modified version of the method of Hedley et al (1983) was applied. The sections were treated with 10 mg $ml^{-1}$ proteinase K (Sigma, St Louis, MO, USA) for 30 min at room temperature and filtered through a 50-μm nylon mesh. The nuclei were treated with 10 mg $ml^{-1}$ RNAase and stained with 25 μg $ml^{-1}$ ethidium bromide (Sigma) for at least 1 h. The DNA was determined by flow cytometry (FACScan; Becton-Dickinson, Mountain View, CA, USA) using 15 mW excitation at 488 nm. The total emission above 560 nm was recorded, and at least 10 000 nuclei from each specimen were analysed. No internal standard was added, as the staining intensity varied from sample to sample. The lowest peak was assigned a DNA index (DI of 1.00), and the DI values of other peaks were calculated using this as a reference. Therefore, possible hypodiploid peaks were identified as diploid and the normal diploid peak as hyperdiploid. Tumours with one peak were considered to be diploid, and those with more than one peak aneuploid. The histograms were interpreted by one of us (SN), unaware of the clinical outcome.

A full peak (G0/G1) coefficient of variation (CV) was calculated, and only samples with a CV of less than 10% were accepted for further analysis. This was done because, in samples with a high CV, a near-diploid peak (DI < 1.1) could remain undetected.

The S-phase fraction was calculated either using the Cellfit program of the FACScan flow cytometer or manually by a modified rectilinear method (Baisch et al, 1975; Camplejohn et al, 1989). The SPF of the stem line with the highest DI was calculated. In cases with different SPF values obtained by the automatic and manual methods, the lower value was chosen. Usually the manual method gave a lower result, because it was applied only in tumours in which the automatic method seemed to give too high values. In these cases, there was usually a skewness to the right of the G1 peak or a noticeable G2 peak of the diploid population within the S-phase of the aneuploid population.

### Statistical analyses

The SPSS-Win program package was used in a PC computer for basic statistical calculations. The statistical tests used are indicated in the results when appropriate. Correlations of categorical variables were examined by contingency tables, which were further analysed by chi-square tests (Pearson correlation coefficient).

Univariate survival analyses were based on the Kaplan–Meier method (log rank analysis; Kaplan and Meier, 1958). Multivariate survival analysis was done with the SPSS-Cox (Cox, 1972) programme package using a forward stepwise procedure and the L ratio significance test. Overall survival analysis included as an event only the deaths resulting from malignant melanoma. Deaths attributable to post-operative complications within 30 days were excluded. Recurrence-free survival (RFS) was defined as the time elapsed between the primary treatment and the recurrent tumour. For all statistical tests, a critical significance level of 5% was chosen. In Cox's multivariate analysis, a removal limit of $P < 0.10$ was used as an additional inclusion criteria.

### RESULTS

There were 191 women (52%) and 178 men (48%) in the cohort. The mean age was 55.5 years (s.d. 15.2) with a range of

*© Cancer Research Campaign 1998*

**Table 2** Association of cell proliferative activity (MAI, M/V index) with clinicopathological parameters.

| Clinicopathological variable | No. of patients | MAI | | | M/V index | | |
|---|---|---|---|---|---|---|---|
| | | ≤ 1 (%) | > 1 (%) | *P* | ≤ 7 (%) | > 7 (%) | *P* |
| Bleeding | 332 | | | <0.000005[a] | | | 0.00002[a] |
| Yes | 94 | 32 | 68 | | 28 | 72 | |
| No | 132 | 65 | 35 | | 58 | 42 | |
| NA[b] | 106 | 57 | 43 | | 52 | 48 | |
| Gender | 332 | | | 0.13 | | | 0.80 |
| Male | 162 | 49 | 51 | | 47 | 53 | |
| Female | 170 | 57 | 43 | | 48 | 52 | |
| Anatomic site | 332 | | | 0.01 | | | 0.01 |
| Head and neck | 54 | 35 | 65 | | 28 | 72 | |
| Trunk and perineum | 161 | 60 | 40 | | 53 | 47 | |
| Upper limbs | 51 | 47 | 53 | | 53 | 47 | |
| Lower limbs | 66 | 55 | 45 | | 46 | 54 | |
| Age at diagnosis | 332 | | | 0.03 | | | 0.02 |
| ≤ 55 years | 153 | 60 | 40 | | 54 | 46 | |
| > 55 years | 179 | 48 | 52 | | 42 | 58 | |
| Co-existing naevus | 332 | | | 0.65[a] | | | 0.34[a] |
| Yes | 153 | 52 | 48 | | 45 | 55 | |
| No | 60 | 50 | 50 | | 43 | 57 | |
| NA[b] | 119 | 56 | 44 | | 53 | 47 | |
| Clark level | 332 | | | <0.000005 | | | <0.000005 |
| I | 8 | 88 | 12 | | 50 | 50 | |
| II | 60 | 88 | 12 | | 72 | 28 | |
| III | 96 | 67 | 33 | | 59 | 41 | |
| IV | 140 | 34 | 66 | | 34 | 66 | |
| V | 28 | 18 | 82 | | 25 | 75 | |
| Tumour thickness (mm) | 316 | | | <0.000005 | | | <0.000005 |
| ≤ 0.75 | 71 | 90 | 10 | | 73 | 27 | |
| 0.76–1.50 | 80 | 69 | 31 | | 63 | 37 | |
| 1.51–4.0 | 115 | 31 | 69 | | 32 | 68 | |
| > 4.0 | 50 | 16 | 84 | | 22 | 78 | |

Numbers in cells indicate percentage of the patients in each category of clinicopathological variables. MAI, mitotic activity index (mitoses/HPF; objective magnification × 40; field diameter, 490 μm). MV index, volume-corrected mitotic index (mitoses $mm^{-2}$ of neoplastic tissue in a section). [a]NA category used as an intermediate category in the analysis; [b]NA, data not available.

19.0–90.0 years. The most common location was the trunk and perineum (47%); 20% were in the lower limbs, 16% in the upper limbs and 17% in the head and neck area.

In the statistical analyses for mitotic rates and SPF, we used the median as the cut-off value (1 for MAI, 7 for M/V index and 4% for SPF respectively). The associations between tumour proliferative activity indicators (MAI, M/V index and SPF) and conventional clinicopathological parameters are shown in Tables 2 and 3. There was a significant association between high (>4%) SPF and high mitotic frequency measured by MAI ($P = 0.002$) and M/V index ($P = 0.001$). The association of MAI with SPF is shown in Table 4.

During the follow-up, 106 patients (29%) had a recurrence, 66 patients (18%) died of melanoma and 46 patients (13%) died of other causes. The crude 5-year survival rate of the patients was 78%. The overall 5-year survival rate of the patients was 85%, and the 5-year RFS rate was 76%.

Clinical, histological and quantitative features predicting RFS and overall survival are shown in Table 5. The most important clinical predictors of RFS in univariate analysis were bleeding and gender. From the histological variables studied, TNM, tumour thickness (Figure 1) and the Clark level of invasion were all highly significant predictors. MAI, M/V index and SPF were the most important quantitative variables predicting RFS. Significant factors predicting overall survival in univariate analysis were gender (Figure 2), bleeding (Figure 3), tumour thickness, Clark level of invasion, TNM category, MAI (Figure 4), M/V index and SPF (Figure 5).

A multivariate Cox analysis was performed on 259 patients with a complete set of data available. It included only the variables that were significant in univariate analysis (gender, bleeding, tumour thickness, Clark level of invasion, MAI, M/V index and SPF). The pT category (consisting of Clark level of invasion and tumour thickness) was excluded from the final model. Tumour thickness ($P = 0.0021$), bleeding ($P = 0.0106$) and high M/V index ($P = 0.0058$) predicted poor recurrence-free survival. High MAI ($P = 0.0002$), bleeding ($P = 0.004$), high SPF (over 4%; $P = 0.009$) and male gender ($P = 0.034$) were statistically significant predictors of poor overall survival (Table 6).

In order to address whether mitotic rates or SPF add significant information to normally available clinical prognosticators, we combined the proliferation markers (MAI, M/V index and SPF) with conventional variables (tumour thickness, bleeding and

© Cancer Research Campaign 1998

**Table 3** Association of SPF with clinicopathological parameters

| Clinicopathological variable | No. of patients | SPF | | |
|---|---|---|---|---|
| | | ≤4% (%) | >4% (%) | *P* |
| Bleeding | 290 | | | 0.05[a] |
| Yes | 77 | 44 | 56 | |
| No | 119 | 61 | 39 | |
| NA[b] | 94 | 51 | 49 | |
| Gender | 290 | | | 0.47 |
| Male | 144 | 56 | 44 | |
| Female | 146 | 52 | 48 | |
| Anatomic site | 290 | | | 0.01 |
| Head and neck | 50 | 38 | 62 | |
| Trunk and perineum | 143 | 59 | 41 | |
| Upper limbs | 42 | 64 | 36 | |
| Lower limbs | 55 | 44 | 56 | |
| Age at diagnosis | 290 | | | 0.19 |
| ≤ 55 years | 130 | 58 | 42 | |
| > 55 years | 160 | 50 | 50 | |
| Co-existing naevus | 290 | | | 0.78[a] |
| Yes | 135 | 53 | 47 | |
| No | 50 | 58 | 42 | |
| NA[b] | 105 | 52 | 48 | |
| Clark level | 290 | | | 0.0007 |
| I | 13 | 77 | 23 | |
| II | 56 | 71 | 29 | |
| III | 78 | 56 | 44 | |
| IV | 119 | 45 | 55 | |
| V | 24 | 29 | 71 | |
| Tumour thickness (mm) | 270 | | | 0.0001 |
| ≤ 0.75 | 64 | 67 | 33 | |
| 0.76–1.50 | 67 | 64 | 36 | |
| 1.51–4.0 | 99 | 45 | 55 | |
| >4.0 | 40 | 27 | 73 | |

Numbers in cells indicate percentage of the patients in each category of clinicopathological variables. SPF, S-phase fraction. [a]NA category used as an intermediate category in the analysis; [b]NA, data not available.

gender) respectively. We also combined MAI and SPF in the same manner. Each of these combinant variables (MAI/Breslow, MAI/bleeding, MAI/gender, MAI/SPF, SPF/Breslow, SPF/bleeding and SPF/gender for overall and M/V index/Breslow and M/V index/bleeding for recurrence-free survival) were used in turn with the other remaining independent variables in a Cox forward stepwise manner.

In overall survival, MAI/bleeding ($P = 0.0035$) and MAI/SPF ($P = 0.0001$) were those variables that clearly added prognostic power to the model. In patients with bleeding tumours, the relative risk of melanoma death was 9.05 (CI 2.1–39.7) in the high MAI (> 1) subgroup compared with the risk of patients with non-bleeding tumours in the low MAI (≤ 1) subgroup. The relative risk of melanoma death for the patients with high MAI (> 1) and high SPF (> 4%) categories was 10.6 (CI 3.2–35.6) compared with the risk of patients with low MAI (≤ 1) and low SPF (≤ 4%) categories (other data not shown). In the recurrence-free survival analysis, the M/V index/Breslow variable was a better prognosticator (overall $P = 0.0002$) than either M/V index or Breslow thickness. The risk of melanoma recurrence between the patients with tumour thickness > 4.0 mm and M/V index > 7 was 38.3 (CI 5–293) compared with the risk of patients with tumour thickness ≤ 0.75 mm and M/V index ≤ 7 (other data not shown).

**Table 4** Association of mitotic activity index (MAI) and S-phase fraction (SPF)

| SPF | MAI | |
|---|---|---|
| | ≤ 1 (number of patients = 142) | > 1 (number of patients = 131) |
| ≤ 4% | 62% | 44% |
| > 4% | 38% | 56% |
| Total | 100% | 100% |

Numbers in cells express percentage of the patients in each MAI category. $\chi^2 = 9.32$; $P = 0.002$. SPF, S-phase fraction; MAI, mitotic activity index (mitoses/HPF, objective magnification × 40, field diameter, 490 μm).

### DISCUSSION

The proliferative activity of cancer cells has a significant prognostic value in several human malignancies (Quinn and Wright, 1990). Mitotic counting is the most commonly used method of assessing proliferative activity in human tumours. The mitotic activity has an independent prognostic value in many human epithelial tumours (Cross and Start, 1996). In malignant melanoma, mitotic counts have been shown to be of prognostic significance in several studies (Schmoeckel and Braun-Falco, 1978; Salman and Rogers, 1990; Evans et al, 1992; Ramsay et al, 1995; Clemente et al, 1996).

The potential value of the mitotic index as a prognostic parameter has been questioned because different authors have obtained varying results (Quinn and Wright, 1990). The variability in fixation, intratumoral heterogeneity, variations in cell size and the criteria for recognition of mitotic figures may all cause interobserver variations (Weidner et al, 1994; Collan et al, 1996; Cross and Start, 1996; Jannink et al, 1996). The reproducibility of the M/V index and MAI have been documented in human tumours (Donhuijsen, 1986; Montironi et al, 1988; Haapasalo et al, 1989b; Lipponen et al, 1990). Jannink et al (1996) found a high degree of intratumour heterogeneity of mitotic activity (MAI and M/V index) in breast cancer. They conclude that multiple blocks should be taken, and the areas with highest proliferation should be selected. According to their results, a correction for the volume percentage of epithelium did not result in remarkable heterogeneity in results between MAI and M/V index. As long as the criteria to assess the mitotic activity are strict, the mitotic index will be reproducible and prognostically relevant.

Flow cytometric analysis of nuclear DNA content and SPF is a feasible method for estimating the malignant potential and growth characteristics of malignant tumours (Seckinger et al, 1989; Keshgegian and Cnaan, 1995). DNA aneuploid primary melanomas recur earlier and more frequently than do DNA diploid ones (von Roenn et al, 1986; Kheir et al, 1988). DNA aneuploidy has also been associated with a shorter survival in primary melanoma (Kheir et al, 1988; Lindholm et al, 1989; Gattuso et al, 1990; Bartkowiak et al, 1991). In our study, DNA ploidy had no impact on survival. Differences in tissue processing, the nuclei measured, DNA histogram/cell cycle analysis and intratumoral heterogeneity may explain the above-mentioned divergent results (Kallioniemi, 1988; Bergers et al, 1996).

 © Cancer Research Campaign 1998

**Table 5** Clinical, histological and quantitative factors related to survival in cutaneous malignant melanoma

| Category (variable) | No. of patients | Recurrence-free 5 years (RFS) (%) | *P*[a] | Surviving at 5 years (%) | *P*[a] |
|---|---|---|---|---|---|
| Gender | | | | | |
| Male | 178 | 75 | 0.0113 | 79 | 0.0056 |
| Female | 191 | 89 | | 89 | |
| Bleeding | | | | | |
| Yes | 98 | 74 | <0.00005[b] | 77 | 0.0002[b] |
| No | 152 | 90 | | 92 | |
| NA[c] | 119 | 79 | | 83 | |
| Clark level | | | | | |
| I | 21 | 94 | <0.00005 | 94 | <0.00005 |
| II | 68 | 98 | | 98 | |
| III | 106 | 90 | | 92 | |
| IV | 145 | 71 | | 76 | |
| V | 29 | 65 | | 65 | |
| Tumour thickness (mm) | | | | | |
| ≤ 0.75 | 82 | 97 | <0.00005 | 97 | <0.00005 |
| 0.76–1.50 | 88 | 90 | | 93 | |
| 1.51–4.0 | 120 | 74 | | 78 | |
| > 4.0 | 50 | 57 | | 63 | |
| TNM category | | | | | |
| pT1–T2,N0,M0 | 174 | 95 | <0.00005 | 96 | <0.00005 |
| pT3,N0.M0 | 139 | 74 | | 78 | |
| pT4,N0,M0 | 56 | 60 | | 65 | |
| MAI | | | | | |
| ≤ 1 | 176 | 92 | <0.00005 | 93 | <0.00005 |
| > 1 | 156 | 69 | | 74 | |
| M/V index | | | | | |
| ≤ 7 | 158 | 91 | <0.00005 | 93 | <0.00005 |
| > 7 | 174 | 72 | | 77 | |
| SPF | | | | | |
| ≤ 4% | 155 | 91 | 0.0001 | 92 | 0.0007 |
| > 4% | 135 | 72 | | 76 | |
| DNA ploidy | | | | | |
| Diploid | 237 | 83 | 0.15 | 85 | 0.25 |
| Aneuploid | 57 | 74 | | 80 | |

[a]Log rank analysis. [b]NA category used as an intermediate category in the analysis. [c]NA, data not available. MAI, mitotic activity index; M/V index, volume-corrected mitotic index; SPF, S-phase fraction.

Like DNA ploidy, SPF has a prognostic value in cutaneous stage I (Bartkowiak et al, 1991) and metastatic (Muhonen et al, 1992) melanoma. Our study supports the important prognostic role of SPF in malignant melanoma. High SPF (over 4%) predicted poor recurrence-free and overall survival in univariate analysis and poor overall survival in multivariate analysis. However, in order to use SPF as a marker of cell proliferative activity, we have to consider that SPF varies considerably in different samples from the same tumour (Kallioniemi, 1988). SPF does not indicate the growth rate of the tumour directly, but merely indicates the proportion of cells synthesizing DNA.

Other cell proliferation markers that can be used on routine tissue sections are Ki-67 antigen, proliferative cell nuclear antigen (PCNA) and silver-binding nucleolar organizer region (AgNOR) staining (Cross and Start, 1996). Ki-67 antigen can be detected with either a polyclonal Ki-67 antibody or a specific monoclonal antibody for Ki-67 epitope (MIB-1; Gerdes et al, 1991). MIB-1 expression correlates with mitotic counts in breast and renal cell carcinoma (Weidner et al, 1994; Cross and Start, 1996; Jochum et al, 1996), but it is liable to the same reproducibility problems as mitotic counts. MIB-1 staining had a prognostic independent value even superior to tumour thickness and mitotic index in primary thick cutaneous melanomas (Ramsay et al, 1995). In addition, immunostaining for Ki-67 antigen is helpful in identifying individuals with thick nodular melanomas who are at risk of metastatic disease (Vogt et al, 1997). PCNA expression in cutaneous melanomas seems to be a marker of tumour progression (Takahashi et al, 1991; Evans et al, 1992), but it may not help in predicting prognosis in these tumours (Reddy et al, 1995). AgNOR counts often correlate with other markers of cell proliferation, but the staining techniques and the counting methods suffer standardization problems (Cross and Start, 1996). So far, AgNOR counting has failed in predicting the prognosis of cutaneous malignant melanoma, and its correlation with other cell proliferation markers in cutaneous melanomas is also controversial (Evans et al, 1992).

In our study, the most important prognostic factors observed in univariate analyses (Table 5) were bleeding of the tumour, gender of the patient, tumour thickness according to Breslow, level of

© Cancer Research Campaign 1998

**Figure 1** Recurrence-free survival according to Breslow thickness in stage I cutaneous malignant melanoma (tumour thickness ≤ 0.75 mm, $n$ = 82; tumour thickness 0.76–1.50 mm, $n$ = 88; tumour thickness 1.51–4.0 mm, $n$ = 120; tumour thickness > 4.0 mm, $n$ = 50; $P$ < 0.00005; $\chi^2$ = 52.67)

**Figure 3** Overall survival according to bleeding in stage I cutaneous malignant melanoma (no bleeding, $n$ = 152; bleeding, $n$ = 98; bleeding unknown, $n$ = 119; $P$ = 0.0002; $\chi^2$ = 17.36)

**Figure 2** Overall survival of women ($n$ = 191) and men ($n$ = 178) in stage I cutaneous malignant melanoma ($P$ = 0.0056; $\chi^2$ = 7.66)

**Figure 4** Overall survival according to MAI in stage I cutaneous malignant melanoma (MAI ≤ 1, $n$ = 176 and MAI > 1, $n$ = 156; $P$ < 0.00005; $\chi^2$ = 31.51)

© Cancer Research Campaign 1998

**Figure 5** Overall survival according to SPF in stage I cutaneous malignant melanoma (SPF ≤ 4%, $n$ = 155 and SPF > 4%, $n$ = 135; $P$ = 0.0007; $\chi^2$ = 11.43)

invasion according to Clark, TNM category and proliferative activity (M/V index, MAI and S-phase fraction). This observation is in agreement with previous studies (Breslow, 1970; Gattuso et al, 1990; Garbe et al, 1995; Straume and Akslen, 1996). In the multivariate analysis, tumour thickness was the best predictor of RFS followed by M/V index and bleeding. The best predictors of overall survival in order of importance were MAI, bleeding, SPF and male gender.

We found a significant association between high mitotic rate and high SPF, and we suggest that SPF measured by FCM from paraffin blocks can be used to predict the aggressiveness of cutaneous malignant melanoma. However, intratumour heterogeneity of DNA ploidy and SPF may interfere with the results when only one tumour sample is analysed (Lipponen et al, 1991; Bergers et al, 1996).

Recently, Kirkwood et al (1996) reported promising results in treating high-risk resected melanoma patients with adjuvant interferon alfa-2b (IFNα-2b). As adjuvant treatments can be toxic and the overall benefits with node-negative patients can be relatively modest, a question arises: are there any subsets of high-risk node-negative patients who would benefit from such treatments? In our study, the combinations of MAI with bleeding, MAI with SPF and M/V index with tumour thickness are variables that add significant information to the normal clinical data set available.

To conclude, MAI and M/V index are strong predictors of the overall and recurrence-free survival of stage I cutaneous malignant melanoma patients in our material. One advantage of the mitotic indices compared with SPF is that no special equipment is needed.

**Table 6** Independent predictors of overall survival and recurrence-free survival in Cox's analysis

| Category | Beta (s.e.) | *P*-value | Hazard rate (95% CI) |
|---|---|---|---|
| *Overall survival* | | | |
| Gender | | | |
| Male | | | |
| Female | –0.63 (0.30) | 0.034 | 0.53 (0.30–0.95) |
| Bleeding | | 0.004 (overall) | |
| No | | | |
| Yes | 1.36 (0.42) | 0.001 | 3.89 (1.71–8.84) |
| NA | 0.76 (0.43) | 0.080 | 2.13 (0.91–4.98) |
| MAI | | | |
| ≤ 1 | | | |
| > 1 | 1.50 (0.40) | 0.0002 | 4.47 (2.05–9.72) |
| SPF | | | |
| ≤ 4% | | | |
| > 4% | 0.82 (0.32) | 0.009 | 2.27 (1.22–4.22) |
| *Recurrence-free survival* | | | |
| Bleeding | | 0.0106 (overall) | |
| No | | | |
| Yes | 0.92 (0.31) | 0.0029 | 2.51 (1.37–4.60) |
| NA | 0.49 (0.32) | 0.13 | 1.63 (0.86–3.07) |
| Tumour thickness (mm) | | 0.0021 (overall) | |
| ≤ 0.75 | | | |
| 0.76–1.50 | 2.07 (0.75) | 0.0057 | 7.97 (1.83–34.69) |
| 1.51–4.0 | 2.33 (0.73) | 0.001 | 10.27 (2.44–43.20) |
| > 4.0 | 2.81 (0.76) | 0.0002 | 16.64 (3.75–73.88) |
| M/V index | | | |
| ≤ 7 | | | |
| > 7 | 0.83 (0.30) | 0.0058 | 2.31 (1.27–4.18) |

Multivariate analysis included 259 patients with a complete set of data available. MAI, mitotic activity index; M/V index, volume-corrected mitotic index; SPF, S-phase fraction; NA, data not available.

© Cancer Research Campaign 1998

However, if available, SPF is also a strong independent prognosticator in stage I cutaneous malignant melanoma. We suggest that tumour proliferation assessed by mitotic rate or SPF, together with conventionally available prognosticators, might be considered as a patient inclusion criteria for further adjuvant treatment trials in node-negative cutaneous malignant melanoma patients.

### ACKNOWLEDGEMENTS

This study has been supported by grants from the Cancer Fund of North Savo, the Culture Fund of Finland and by EVO funding from Kuopio University Hospital. The skilful technical assistance of Ms Monica Schoultz and statistical assistance of Mrs Pirjo Halonen is acknowledged.

### REFERENCES

Baisch H, Göhde W and Linden WA (1975) Analysis of PCP-data to determine the fraction of cells in the various phases of cell cycle. *Radiat Environ Biophys* **12**: 31–39

Barnhill RL, Fine JA, Roush GC and Berwick M (1996) Predicting five-year outcome for patients with cutaneous melanoma in a population-based study. *Cancer* **78**: 427–432

Bartkowiak D, Schumann J, Otto FJ, Lippold A and Drepper H (1991) DNA flow cytometry in the prognosis of primary malignant melanoma. *Oncology* **48**: 39–43

Bergers E, van Diest PJ and Baak JPA (1996) Tumour heterogeneity of DNA cell cycle variables in breast cancer measured by flow cytometry. *J Clin Pathol* **49**: 931–937

Breslow A (1970) Thickness, cross-sectional areas and depth of invasion in the prognosis of cutaneous melanoma. *Ann Surg* **12**: 902–908

Camplejohn R, MacCartney J and Morris R (1989) Measurement of S-Phase fractions in lymphoid tissue comparing fresh versus paraffin-embedded tissue and 4′,6′-diamidino-2-phenylindole dihydrochloride versus propidium iodide staining. *Cytometry* **10**: 410–416

Clark WH Jr, From L, Bernardino EA and Mihm MC (1969) The histogenesis and biologic behaviour of primary human malignant melanomas of the skin. *Cancer Res* **29**: 705–726

Clemente C, Mihm M, Bufalino R, Zurrida S, Collini P and Cascinelli N (1996) Prognostic value of tumor infiltrating lymphocytes in the vertical growth phase of primary cutaneous melanoma. *Cancer* **77**: 1303–1310

Collan YUI, Kuopio T, Baak JPA, Becker R, Bogomoletz WV, Deverell M, van Diest P, van Galen C, Gilchrist K, Javed A, Kosma V-M, Kujari H, Luzi P, Mariuzzi GM, Matze E, Montironi R, Scarpelli M, Sierra D, Sisti S, Toikkanen S, Tosi P, Whimster WF and Wisse E (1996) Standardized mitotic counts in breast cancer; evaluation of the method. *Pathol Res Pract* **192**: 931–941

Cox DR (1972) Regression models and life tables with discussion. *J Stat Soc B* **34**: 187–192

Cross SS and Start RD (1996) Estimating mitotic activity in tumours. *Histopathology* **29**: 485–488

Donhuijsen K (1986) Mitosis counts: reproducibility and significance in grading of malignancy. *Hum Pathol* **17**: 1122–1125

Eastwood J and Baker TG (1984) Cutaneous malignant melanoma in West Yorkshire. II. A prospective study of recurrence and prediction of lymph nodal metastasis. *Br J Cancer* **50**: 35–43

Eskelinen MJ, Lipponen PK, Papinaho S, Aaltomaa S, Kosma V-M, Klemi P and Syrjänen K (1992) DNA flow cytometry, nuclear morphometry, mitotic indices and steroid receptors as independent prognostic factors in female breast cancer. *Int J Cancer* **51**: 555–561

Evans AT, Blessing K, Orrell JM and Grant A (1992) Mitotic indices, anti-PCNA immunostaining, and AgNORs in thick cutaneous melanomas displaying paradoxical behaviour. *J Pathol* **168**: 15–22

Friedlander ML, Hedley DW and Taylor IW (1984) Clinical and biological significance of aneuploidy in human tumours. *J Clin Pathol* **37**: 961–974

Garbe C, Buttner B, Bertz J, Burg G, d'Hoedt B, Drepper H, Guggenmoos-Holzmann I, Lechner W, Lippold A, Orfanos CE, Peters A, Rassner G, Stadler R and Stroebel W (1995) Primary cutaneous melanoma. Identification of prognostic groups and estimation of individual prognosis for 5093 patients. *Cancer* **75**: 2484–2491

Gattuso P, Reddy V, Solans E, Kathuria S, Aranha GV, Jacobs HK and Walloch J (1990) Is DNA ploidy of prognostic significance in stage I cutaneous melanoma? *Surgery* **108**: 702–708; discussion 708–709

Gerdes J, Li L, Schlueter C, Duchrow M, Wohlenberg C, Gerlach C, Kloth S, Brandt E and Flad HD (1991) Immunobiochemical and molecular biologic characterization of the cell proliferation-associated nuclear antigen that is defined by monoclonal antibody Ki-67. *Am J Pathol* **138**: 867–873

Haapasalo H, Pesonen E and Collan Y (1989a) Volume-corrected mitotic index (M/V-index). The standard of mitotic activity in neoplasms. *Pathol Res Pract* **185**: 551–554

Haapasalo H, Collan Y, Atkin N and Seppä A (1989b) Prognosis of ovarian carcinomas: prediction by histoquantitative methods. *Histopathology* **15**: 167–178

Hedley DW, Friedlander ML, Taylor IW, Rugg CA and Musgrove EA (1983) Method for analysis of cellular DNA content of paraffin-embedded pathological material using flow cytometry. *J Histochem Cytochem* **31**: 1333–1335

Jannink I, Risberg B, van Diest PJ and Baak JPA (1996) Heterogeneity of mitotic activity in breast cancer. *Histopathology* **29**: 421–428

Jochum W, Schröder S, Al-Taha R, August C, Gross A, Berger J and Padberg B-C (1996) Prognostic significance of nuclear DNA content and proliferative activity in renal cell carcinomas. A clinicopathological study of 58 patients using mitotic count, MIB-1 staining and DNA cytophotometry. *Cancer* **77**: 514–521

Kaplan EL and Meier P (1958) Nonparametric estimation from incomplete observations. *J Am Stat Assoc* **53**: 457–481

Kallioniemi OP (1988) DNA flow cytometry in oncology – methodology and prognostic value in breast and ovarian cancer. *Cytometry* **9**: 164–169

Keshgegian AA and Cnaan A (1995) Proliferation markers in breast carcinoma. Mitotic figure count, S-phase fraction, proliferating cell nuclear antigen, Ki-67 and MIB-1. *Am J Clin Pathol* **104**: 42–49

Kheir SM, Bines SD, von Roenn JH, Soong S-J and Coon JS (1988) Prognostic significance of DNA aneuploidy in stage I cutaneous melanoma. *Ann Surg* **207**: 455–461

Kirkwood JM, Strawderman MH, Ernstoff MS, Smith TJ, Borden EC and Blum RH (1996) Interferon alfa-2b adjuvant therapy of high-risk resected cutaneous melanoma: the eastern cooperative oncology group trial EST 1684. *J Clin Oncol* **14**: 7–17

Lindholm C, Hofer P, Jonsson H and Tribukait B (1989) Flow DNA-cytometric findings of paraffin embedded primary cutaneous melanomas related to prognosis. *Virchows Arch* B **58**: 147–151

Lipponen PK, Kosma V-M, Collan Y, Kulju T, Kosunen O and Eskelinen MJ (1990) Potential of nuclear morphometry and volume corrected mitotic index in grading transitional-cell carcinoma of the urinary bladder. *Eur J Urol* **17**: 333–337

Lipponen PK, Eskelinen MJ and Nordling S (1991) Intratumoral heterogeneity of DNA indexes in transitional cell bladder cancer: relation to tumour histology. *Eur Urol* **20**: 311–314

Montironi R, Collan Y, Scarpelli M, Sisti S, Barbatelli G, Carnevali A, Pisani E and Mariuzzi GM (1988) Reproducibility of mitotic counts and identification of mitotic figures in malignant glial tumours. *Appl Pathol* **6**: 258–265

Muhonen T, Pyrhönen S, Laasonen A, Wasenius V-M, Asko-Seljavaara S, Franssila K and Kangas L (1992) Tumour growth rate and DNA flow cytometry parameters as prognostic factors in metastatic melanoma. *Br J Cancer* **66**: 528–532

Quinn CM and Wright NA (1990) The clinical assessment of proliferation and growth in human tumours: evaluation of methods and applications as prognostic variables. *J Pathol* **160**: 93–102

Ramsay JA, From L, Iscoe NA and Kahn HJ (1995) MIB-1 proliferative activity is a significant prognostic factor in primary thick cutaneous melanomas. *J Invest Dermatol* **105**: 22–26

Reddy VB, Gattuso P, Aranha G and Carson HJ (1995) Cell proliferation markers in predicting metastases in malignant melanoma. *J Cutan Pathol* **22**: 248–251

Salman SM and Rogers GS (1990) Prognostic factors in thin cutaneous malignant melanoma. *J Dermatol Surg Oncol* **16**: 413–418

Schmoeckel C and Braun-Falco O (1978) Prognostic index in malignant melanoma. *Arch Dermatol* **114**: 871–873

Seckinger D, Sugarbaker E and Frankfurt O (1989) DNA content in human cancer. *Arch Pathol Lab Med* **113**: 619–626

Straume O and Akslen LA (1996) Independent prognostic importance of vascular invasion in nodular melanomas. *Cancer* **78**: 1211–1219

Takahashi H, Strutton GM and Parsons PG (1991) Determination of proliferating fractions in malignant melanomas by anti-PCNA/cyclin monoclonal antibody. *Histopathology* **18**: 221–227

 © Cancer Research Campaign 1998

Talve L, Kainu J, Collan Y and Ekfors T (1996) Immunohistochemical expression of p53 protein, mitotic index and nuclear morphometry in primary malignant melanoma of the skin. *Pathol Res Pract* **192**: 825–833

UICC (1987) *TNM Classification of Malignant Tumours*. Fourth, fully revised edition. Berlin, Springer-Verlag, pp. 88–90

Vogt T, Zipperer K-H, Vogt A, Hölzel D, Landthaler M and Stolz W (1997) p53 protein and Ki-67 antigen expression are both reliable biomarkers of prognosis in thick stage I nodular melanomas of the skin. *Histopathology* **30**: 57–63

von Roenn JH, Kheir SM, Wolter JM and Coon JS (1986) Significance of DNA abnormalities in primary malignant melanoma and nevi: a retrospective flow cytometric study. *Cancer Res* **46**: 3192–3195

Weidner N, Moore DH and Vartanian R (1994) Correlation of Ki-67 antigen expression with mitotic figure index and tumor grade in breast carcinomas using the novel 'paraffin'-reactive MIB-1 antibody. *Hum Pathol* **25**: 337–342

© Cancer Research Campaign 1998